# European Hares, *Lepus europaeus*, Represent a Reservoir Host for *Thelazia callipaeda* in Romania

**DOI:** 10.3390/pathogens11111225

**Published:** 2022-10-24

**Authors:** Vlad-Dan Cotuțiu, Andrei Daniel Mihalca, Katarzyna Anna Hołówka, Angela Monica Ionică, Cristina Daniela Cazan, Călin Mircea Gherman

**Affiliations:** 1 Department of Parasitology and Parasitic Diseases, University of Agricultural Sciences and Veterinary Medicine of Cluj-Napoca, Calea Mănăștur 3-5, 400372 Cluj-Napoca, Romania; 2 Molecular Biology and Veterinary Parasitology Unit (CDS-9), University of Agricultural Sciences and Veterinary Medicine of Cluj-Napoca, Calea Mănăștur 3-5, 400372 Cluj-Napoca, Romania; 3 Clinical Hospital of Infectious Diseases of Cluj-Napoca, Iuliu Moldovan Street Nr. 23, 400003 Cluj-Napoca, Romania

**Keywords:** *Thelazia callipaeda*, *Lepus europaeus*, reservoir host, Thelaziosis

## Abstract

Thelaziosis caused by *Thelazia callipaeda* is an emerging disease in Europe. Only two reports of naturally infected lagomorphs have been published so far. The aim of this study was to evaluate the status of the Romanian populations of European brown hares, *Lepus europaeus* as reservoir hosts for *T. callipaeda*. Between November 2019 and November 2021, the eyes of 326 *L. europaeus* carcasses were examined for the presence of ocular parasites. Nematodes were stored in plastic vials with physiological saline, followed by morphological and molecular identification. QGis 3.20 and EpiInfo^TM^ 7 were used for mapping and statistical analysis. Four (1.23%) hares harbored *T. callipaeda* infection, with a total of 84 nematodes collected (mean intensity 21 nematodes/host), with 45 males, 39 females (two sexually immature, seven with only eggs, and 30 with eggs and larvae). One specimen from each host was successfully sequenced resulting in a 100% similarity with several other sequences of *T. callipaeda* haplotype 1. Statistical analysis revealed no significant results. The current study represents a first report of *T. callipaeda* in the European brown hare in Romania, and the second in Europe, also reiterating the role of lagomorphs as reservoir hosts for this zoonotic ocular nematode.

## 1. Introduction

Thelaziosis caused by *Thelazia callipaeda* (Spirurida, Thelaziidae) is a rapidly emerging zoonosis reported across most of Europe and Asia [1]. Domestic and wild carnivores are considered the primary vertebrate hosts of *T. callipaeda* [2]. Still, occasionally, adult nematodes were reported from other mammals such as other carnivores, lagomorphs, wild boars, and humans [3,4,5,6,7].

Infections in both domestic and wild carnivores are commonly reported across the distribution range of this nematode [1]. Human ocular infections follow an emerging trend in most countries where *T. callipaeda* had been reported in the main reservoir hosts [6,7,8]. These findings not only underline the zoonotic potential of this nematode but also highlight the clinical implications of the disease. Symptoms range from mild to severe conjunctivitis [9], further complicated by bacterial or fungal infections, which may lead to corneal ulcers [7].

In Romania, the disease was first diagnosed in 2014 [10], in a domestic dog from the western part of the country. Subsequent surveillance documented the spread across most of Romania’s territory, in a wide variety of hosts: domestic dogs [11,12,13], domestic cats [13], jackals, wolves, wildcats [14], foxes [15], and mustelids [16]. However, despite its wide distribution in animals, no human cases have been documented in Romania, so far. Although lagomorphs (hares, rabbits) are known as suitable hosts for *T. callipaeda* [3,4], there are no studies or reports of these hosts in Romania. Hence, we aimed to investigate the presence of *T. callipaeda* in hares, *Lepus europaeus* collected in various regions of Romania, and to evaluate their reservoir role.

## 2. Results

**Figure 1:**
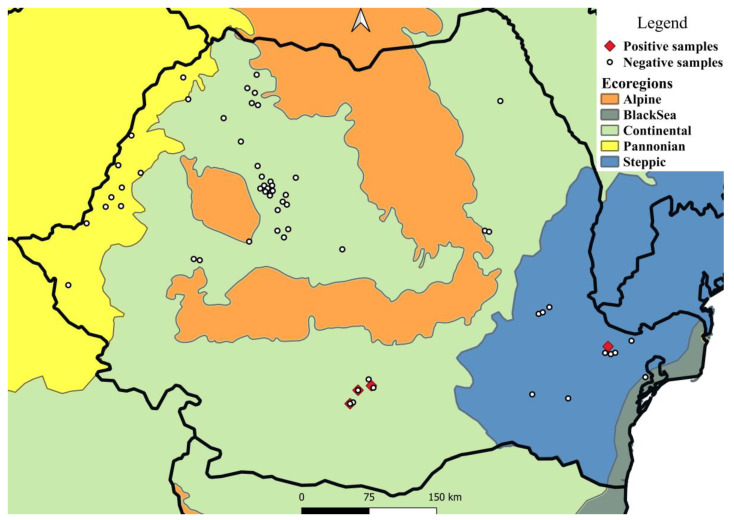
Sampling areas and distribution of *T. callipaeda* in European brown hares from Romania, by ecoregion.

**Figure 2:**
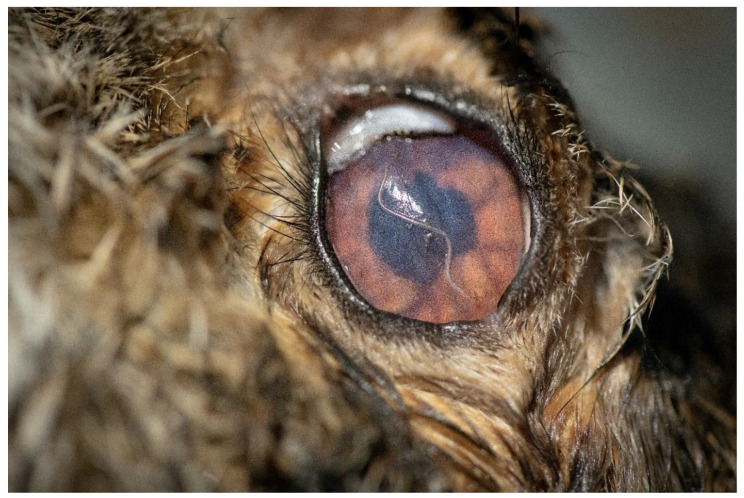
*L. europaeus* with a specimen of *T. callipaeda* present in its left eye.

Four of the 326 European brown hares examined were positive for ocular nematodes (1.23%) (Table 1, Figure 1 and Figure 2). A total of 84 nematodes were collected. All nematodes were morphologically identified as *T. callipaeda*. The intensity varied between 1 and 70 nematodes/hare, with a mean intensity of 21 (Table 2). Seven (18.9%) of the 37 mature females presented non-blastomerized eggs and blastomerized eggs, whereas 30 (81.1%) also presented larvated eggs as well as larvae inside the uterus.

All four specimens selected for molecular analysis were successfully sequenced, showing a 100% similarity with several sequences of *T. callipaeda* haplotype 1 (GenBank: MK546436- MK546439, MF578281; MG913802; AP017700; OM470911).

Statistical analysis, using Pearson’s chi-squared test, correlating sex (*p* = 0.6563), ecoregion (*p* = 0.1171), altitude intervals (*p* = 0.1721) to infection status revealed no significant results, the *p* values exceeding the 0.05 benchmark.

## 3. Discussion

The current study serves as the first report of *T. callipaeda* in European brown hares in Romania and the second in Europe [3]. Moreover, the presence of larvae in the adult females of *T. callipaeda* is a strong indicator that hares are definitive (final) and also reservoir hosts, being able to transmit the infection in natural conditions. However, due to the low prevalence, we cannot suggest that hares are significant reservoir hosts in Romania, mainly as the area is known as hypernedmic for *T. callipaeda* in red foxes [15]. For instance, in Italy, the prevalence in hares was significantly higher (3/13, 23.1%) [3]. The higher prevalence in foxes could be linked to their crepuscular activity, which fits the one of *Phortica variegata* [17], whereas European brown hares are predominantly crepuscular and nocturnal, with a peak in the late afternoon, during the mating season, in spring [18,19]. Additionally, two wild European rabbits, *Oryctolagus cuniculus* from Portugal, also harbored *T. callipaeda* infection [4], highlighting the susceptibility of European lagomorphs to infection with this nematode.

Lagomorphs were only reported as natural hosts for *T. callipaeda* in Europe and Russia [3,4,20]. An experimental study, in Russia, concluded that the estimated life span of sexually mature forms of *T. callipaeda* in laboratory rabbits, *O. cuniculus* is of around six months [21], which, under natural conditions, could overlap with the activity period of the vectors. Moreover, the shallow burrowing behavior of brown hares during their inactive periods might leave them exposed to the activity of *P. variegata* [22].

The potential susceptibility of European brown hares as feeding hosts for *Phortica variegata* was suggested by Otranto et al. [3]. Interestingly, landscape diversity for the European hare has seen a shift towards woodlands, brushes, unimproved grasslands, and field margins [23], potentially making them more susceptible both to predators [24] and diseases. This follows the trends of the decline of farmland biodiversity, which can be attributed to the intensification of the agricultural industry in the late 20th century in Europe, according to several studies on agri-environment schemes [25,26,27].

Because of the scarcity of hare carcass availability outside of the hunting season, which takes place between November and January, the current study is mainly limited to mature and immature adult stages of *T. callipaeda*. Subsequently, any larval stages present during the peak infestation season March–June [28] will have either matured or died, affecting both the overall prevalence rate as well as the intensity within the host.

Therefore, there is an increasing need to determine the complexity of the sylvatic cycle and the diversity of reservoir hosts in relation to their ecology to better understand and implement preventive measures for limiting this zoonotic disease. This latter aspect has become a key point over the past ten years, as more human cases have emerged worldwide [6,7,8].

## 4. Materials and Methods

Between November 2019 and November 2021, 326 carcasses of European hares, *Lepus europaeus*, were examined as part of a broader survey of their parasites (unpublished data). Of these, six were collected outside of the November–February period, whereas another eight had an unknown collection date (Table 1). The study area comprised 17 counties covering four ecoregions (Figure 1). The carcasses originated from legally hunted individuals or roadkills. The following information was recorded for each animal: date and location of collection, and sex.

As part of the necropsy procedure, the eyes of each carcass were examined under a stereo zoom microscope, with the lateral and medial canthus dissected to uncover the entire globe. Upon detection, nematodes were placed in a vial with physiological saline (0.9%), followed by a morphological examination.

Morphological identification of the ocular nematodes was performed using the keys provided by [20,29], during which each nematode’s sex and developmental stages were recorded. Intact specimens underwent detailed morphometric analysis following preservation in 4% formalin solution. All measurements were done using an Olympus microscope (Olympus BX61) and dedicated software.

One randomly selected nematode from each brown hare was stored in 70% ethanol and used for molecular characterization. DNA extraction was performed individually from each nematode using the ISOLATE II Genomic DNA Kit (Bioline Meridian Bioscience, Luckenwalde, Germany), according to the manufacturer’s instructions, and stored at −20 °C until further use. The samples were processed by PCR amplification of a 670-bp gene region the cytochrome c oxidase subunit 1 (cox 1), using a C1000™ Thermal Cycler (Bio-Rad, London, UK) and the NTF/NTR primer pair, as previously described [30]. Sequencing was performed by Macrogen Europe (Amsterdam, The Netherlands), while the assembled chromatograms and consensus sequences were translated and edited using the Geneious 4.8.5 software (Biomatter Ltd., Auckland, New Zealand). Lastly, using the Basic Local Alignment Search Tool (BLAST), the consensus sequences were compared with the available data in the GenBank^®^ database.

Mapping was performed using the free open source QGis Geographic Information System (version 3.20 Odense, QGis Development Team, 2021), including ecoregions as a layer. Statistical analysis was performed using the EpiInfo^TM^ 7 software (CDC, Atlanta, GA, USA, 2021), recording and calculating values for the frequency, prevalence, as well as 95% confidence interval of infestation, according to several parameters (Table 1).

## 5. Conclusions

The current study represents the first report of the presence of *T. callipaeda* in European brown hares in Romania, while also emphasizing the role as a reservoir host of lagomorphs for the aforementioned nematode.

## Figures and Tables

**Table 1:** Sampled European brown hares according to the sex, altitude, and ecoregion.

Variable		Sampled	Positive	Prevalence (%)	95% CI
Sex	Males	132	2	1.52	0.42–5.36
	Females	136	2	1.47	0.4–5.2
	Undetermined	58	0	0	0–6.21
Altitude interval (meters)	0–50	15	1	6.66	1.19–29.82
	51–100	147	0	0	0–2.55
	101–200	50	0	0	0–7.13
	201–300	63	3	4.76	1.63–13.09
	301–500	41	0	0	0–8.57
	≥501	10	0	0	0–27.75
Ecoregion	Pannonian	161	0	0	0–2.33
	Continental	146	3	2.05	0.7–5.87
	Alpine	2	0	0	0–65.76
	Steppic	17	1	5.88	1.05–26.98
	Pontic	0	0	0	0
Sample season	Winter	227	4	1.76	0.69–4.44
	Spring	5	0	0	0–43.45
	Summer	1	0	0	0–79.35
	Autumn	85	0	0	0–4.32
	Unknown	8	0	0	0–32.44
Total		326	4	1.23	0.48–3.11

**Table 2:** *Thelazia callipaeda* infection intensity and population structure in European brown hares from Romania.

Positive Animals	*T. callipaeda* Collected	Infestation Type	Left Eye Intensity	Right Eye Intensity	*T. callipaeda* Population Structure		
					Male	Female	L5 Female
Hare 1	1	Unilateral	1	0	0	0	1
Hare 2	4	Unilateral	4	0	2	2	0
Hare 3	9	Bilateral	1	8	2	6	1
Hare 4	70	Bilateral	45	25	41	29	0
Median	6.5		2.5	4	2	4	0.5

## Data Availability

The data generated or analyzed during this study is included in this published article.
